# What are the social determinants of road traffic death in global context?

**Published:** 2019-08

**Authors:** Toktam Paykani

**Affiliations:** ^*a*^Social Development and Health Promotion Research Center, Gonabad University of Medical Science, Gonabad, Iran.

## Abstract

**Background:**

The World Health Organization (WHO) estimates that road accidents cause over 1.3 million deaths per year. Considering WHO conceptual framework for action on the social determinants of health (SDH), this study aim to answer the question of why some countries have high levels of deaths due to road traffic crashes, and some have low levels.

**Methods:**

Qualitative comparative analysis using fuzzy sets (fsQCA) was performed to identify the structural conditions linking to road traffic death across 123 countries from all WHO regions. The outcome was 2016 reported number of road traffic deaths. The potential causal conditions according to the SDH conceptual framework were level of country economic development (GDP), urban population (% of total), availability of health-system resources and income inequality. The data at country level were obtained from the different international data sources, during 2015-2017.

**Results:**

The fsQCA truth table analysis revealed one pathway to low-level road traffic death that was low-level income inequality combined with high-level health system resources and high-level GDP. This pathway specified a combination of structural conditions that is sufficient to explain low-level road traffic death.

On the other hand, the fsQCA truth table analysis indicated three pathways to high-level road traffic death; (1) low-level urbanization combined with low-level health system resources and low-level GDP, (2) high-level income inequality and low-level urbanization and low-level GDP (3) high-level income inequality combined with high-level urbanization and low-level health system resources. These configurations specified the combination of structural conditions that were sufficient to explain high-level road traffic death.

The case of IRAN with a relatively high level of economic development (fuzzy score=0.65), low level of health-system resources (fuzzy score=0.1), a high level of urbanization (fuzzy score=0.85) and high level of inequality (fuzzy score=1) indicated a configuration linked to the set of countries with high rates of deaths from road accidents in the world (fuzzy score=0.82).

**Conclusions:**

The structural determinants and conditions of daily life that highlighted by WHO commission on social determinants of health may responsible for a part of inequalities in death from road traffic accidents.

**Keywords:**

QCA, Road traffic death, Social Determinants of Health, Inequality

